# Evaluation of Complexation Ability Using a Sensor Electrode Chip Equipped with a Wireless Screening System

**DOI:** 10.3390/s120608405

**Published:** 2012-06-19

**Authors:** Takaaki Isoda, Ikuko Urushibara, Hikaru Sato, Noriyoshi Yamauchi

**Affiliations:** 1 Department of Life and Environment Engineering, Faculty of Environmental Engineering, University of Kitakyushu, 1-1, Hibikino, Wakamatsu, Kitakyushu 808-0135, Japan; 2 AR'S CO., Ltd., 5-1, Yokohama Creation Square, Sakaecho, Kanagawa, Yokohama 221-0052, Japan; 3 The Graduate School of Information, Production and Systems, Waseda University, 2-7, Hibikino, Wakamatsu, Kitakyushu 808-0135, Japan

**Keywords:** chemical adsorption, electrode, chelate, metal ion

## Abstract

We fabricated an electrode chip with a structure coated by an insulation layer that contains dispersed SiO_2_ adsorbent particles modified by an amino-group on a source-drain electrode. Voltage changes caused by chelate molecule adsorption onto electrode surfaces and by specific cation interactions were investigated. The detection of specific cations without the presence of chelate molecules on the free electrode was also examined. By comparing both sets of results the complexation ability of the studied chelate molecules onto the electrode was evaluated. Five pairs of source-drain electrodes(×8 arrays) were fabricated on a glass substrate of 20 × 30mm in size. The individual Au/Cr (1.0/0.1μm thickness) electrodes had widths of 50 μm and an inter-electrode interval of 100μm.The fabricated source-drain electrodes were further coated with an insulation layer comprising a porous SiO_2_ particle modified amino-group to adsorb the chelate molecules. The electrode chip was equipped with a handy-type sensor signal analyzer that was mounted on an amplifier circuit using a Miniship™ or a system in a packaged LSI device. For electrode surfaces containing different adsorbed chelate molecules an increase in the sensor voltage depended on a combination of host-guest reactions and generally decreased in the following order:5,10,15,20-tetrakis(*N*-methylpyridinium-4-yl)-21*H*,23*H*-porphine, tetrakis(*p*-toluenesulfonate) (TMPyP)as a Cu^2+^chelator and Cu^2+^>2-nitroso-5-[*N*-*n*-propyl-*N*-(3-sulfopropyl)amino]phenol(nitroso-PSAP) as an Fe^2+^chelator and Fe^2+^>4,7-diphenyl-1,10-phenanthrolinedisulfonic acid, disodium salt (BPDSA) as an Fe^2+^chelatorand Fe^2+^>3-[3-(2,4-dimethylphenylcarbamoyl)-2-hydroxynaphthalene-1-yl-azo]-4-hydroxybenzenesulfonic acid, sodium salt (XB-1) as a Mg^2+^chelator and Mg^2+^>2,9-dimethyl-4,7-diphenyl-1,10-phenanthrolinedisulfonic acid, disodium salt (BCIDSA) as a Cu^2+^chelator and Cu^2+^, respectively. In contrast, for the electrode surfaces with adsorbed *O,O*′-bis(2-aminoethyl)ethyleneglycol-*N*,*N*,*N*′,*N*′-tetraacetic acid (GEDTA) or *O*,*O*′-bis(2-aminophenyl)ethyleneglycol-*N*,*N*,*N*′,*N*′-tetraacetic acid, tetrapotassium salt, hydrate (BAPTA) as a Ca^2+^chelator no increase in the detection voltage was found for all the electrode tests conducted in the presence of Ca^2+^.To determine the differences in electrode detection, molecular orbital (MO) calculations of the chelate molecules and surface molecular modeling of the adsorbents were carried out. In accordance with frontier orbital theory, the lowest unoccupied MO (LUMO) of the chelate molecules can accept two lone pair electrons at the highest occupied MO (HOMO) of the amino group on the model surface structure of the SiO_2_ particle. As a result, a good correlation was obtained between the LUMO-HOMO difference and the ion response of all the electrodes tested. Based on the results obtained, the order of adsorbed chelate molecules on adsorption particles reflects the different metal ion detection abilities of the electrode chips.

## 1. Introduction

Recently, the quartz crystal microbalance (QCM) method and the surface plasmon resonance (SPR) method have been developed to analyze the host-guest interactions of molecules based on chemical adsorption. The concentration limits of the target substances range from ng/mL to pg/mL. As an example of a QCM application, a QCM surface within a flow apparatus has been reported to monitor heavy metal ions in aqueous solutions. These polymer grafted QCM surface selectively adsorb heavy metal ions such as Cu, Pb, Cr and Cd [1]. By measuring the binding interaction of 8-hydroxy-2&prime-deoxyguanosine (8-OHdG), which is anoxidative DNA lesion resulting from reactive oxygen species, a modified QCM surface that functions by iron complex interactions has been reported [2]. Interestingly, SPR examples have also been reported for immunosensing using a gold surface modified with a thin polyion complex film to suppress non-specific protein adsorption [3].An example is the measurement of serum protein adsorption onto the surface of interdigitated gold surface coated with a series of hydroxyl and methyl functionalized thiols [4].

On the other hand, the sensitivity of an electrode sensor is inferior to SPR and the QCM method although there is an advantage to reducing the size of a sensor by micro-electromechanical system (MEMS) technology. For example, an amperometric method for the determination of copper ions has been developed using disposable, screen-printed electrodes modified with the ligand bis-cyclohexanone oxaldihydrazone [5]. The electrodes gave a peak at +250 mV *vs*. the SCE and a linear response was obtained for copper from 0.03–0.3 mM (r = 0.983, n = 13).Phytase immobilization on modified electrodes for amperometric detecting has also been reported. This electrode detected the phytic acid contained in seeds of grains and vegetables with a detection limit of 0.19 mmol·L^−1^[6].The miniaturization of equipment is also a great advantage because a direct combination with amplification circuits is possible. For example, the development of a taste analysis for the trace assessment of metal ions has been reported[7]. The detection system was based on modified electrodes and a chelating agent that was able to capture metal ions at very low concentrations. The system was able to differentiate between eight metal ions (Al^3+^, Fe^3+^, Cd^2+^, Pb^2+^, Hg^2+^, Cu^2+^, Ca^2+^ and Ag^+^) at micromolar levels in ultrapure water. In a similar example, a multi-electrode array based on coated-film electrodes has been developed[8]. The multi-electrode array contained pH, K^+^, Na^+^ and Ca^2+^ sensors and showed excellent electrode properties that were found to be comparable to commercial ion-selective electrodes. As an ultimate example, the fabrication and characterization of a MEMS-based lab-on-a-chip system for measuring Ca^2+^ ion concentrations and currents around single cells has been reported [9].However, the measurement of chemical adsorption with QCM and SPR using an electrode sensor method has not been achieved, although recent electrode detections have shown adequate performance as a sensing tool.

We previously reported on a chip-mounted microelectrode composed of a source- and drain-electrodes coated with an insulator layer and adsorbent particles to immobilize antibody molecules. It was investigated for the detection of antigens in a phosphoric acid buffer solution(PBS)[10,11].The source-drain electrode was fabricated on a chip and the insulator layer was composed of a novolac resin that was deposited onto SiO_2_ particles as the adsorbent on the electrode pair. Antibody molecules were quickly immobilized onto the SiO_2_ particles by various adsorption phenomena. When a liquid is deposited onto the surface of an insulator an electric double layer arises at the boundary surface. Here, the insulator molecules become polarized, resulting in opposing charges on the boundary surface between the liquid and the solid, as well as on the opposite side of the insulator. In the case of a solution containing antigen molecules that deposit onto the surface of an insulator with immobilized antibodies, specific antigen and antibody reactions occur causing the charge at the reverse side of the insulator to increase. Based on this principle, a source-drain electrode produces a signal in response to changes in the current originating from the antigen-antibody reactions at the boundary surface of the insulator layer.

However, this measurement has a problem in that repeatability is poor. This is because the sensitivity of an antigen-antibody reaction depends on the amount of antibodies absorbed onto the surface of the SiO_2_ particles. The electrode chip surface is required to quickly achieve maximum adsorption of the host molecule onto the SiO_2_ particles. Detailed research is required about the chemical structure of the host molecule to determine the interaction requirements on the surface of the SiO_2_ particles. Therefore, we investigated chelates as model compounds for the host molecule. The molecular weights of the chelate molecules are lower than those of antibodies and the requirements of the chemical structure are obvious. A chelate molecule reacts with a specific cation. The change in voltage caused by the chelation reaction indicates chelate molecule adsorption onto the surface of the SiO_2_ particles.

In this study, an electrode chip with an insulation layer coating dispersed with SiO_2_ adsorbent particles modified by an amino-group on the source-drain electrode was fabricated. A change in voltage because of various chelate molecules adsorbed onto SiO_2_ adsorbent particles and because of specific cations was investigated. In the same way, the detection performance for specific cations by an electrode without chelate molecules was also examined. By comparing both sets of results the complexation ability of the chelate compounds by these electrode sensors was also evaluated.

Five pairs of source-drain electrodes (×8 arrays) were fabricated on a glass substrate20×30mm in size and the individual Au/Cr (1.0/0.1μm thickness) electrodes had widths of 50 μm and an inter-electrode interval of 100μm.The fabricated source-drain electrodes were further coated with an insulation layer comprising porous SiO_2_ particles to adsorb the chelate molecules. Seven types of commercial chelate molecules were examined and the detection voltage of specific metal ions was evaluated before and after the adsorption of chelate molecules onto the electrode. To clarify the differences in electrode detection, as well as the difference in interaction between a chelate molecule and the surface of the adsorbent, molecular orbital (MO) calculations of the chelate molecules were carried out and a surface molecular structure model of the adsorbent was compiled. Based on the results, the detection mechanism of the adsorbed chelate molecules with specific metal ions is discussed.

## 2. Experiments

### 2.1.Detection Principle of the Adsorbed Chelate Molecules

**Figure 1.**
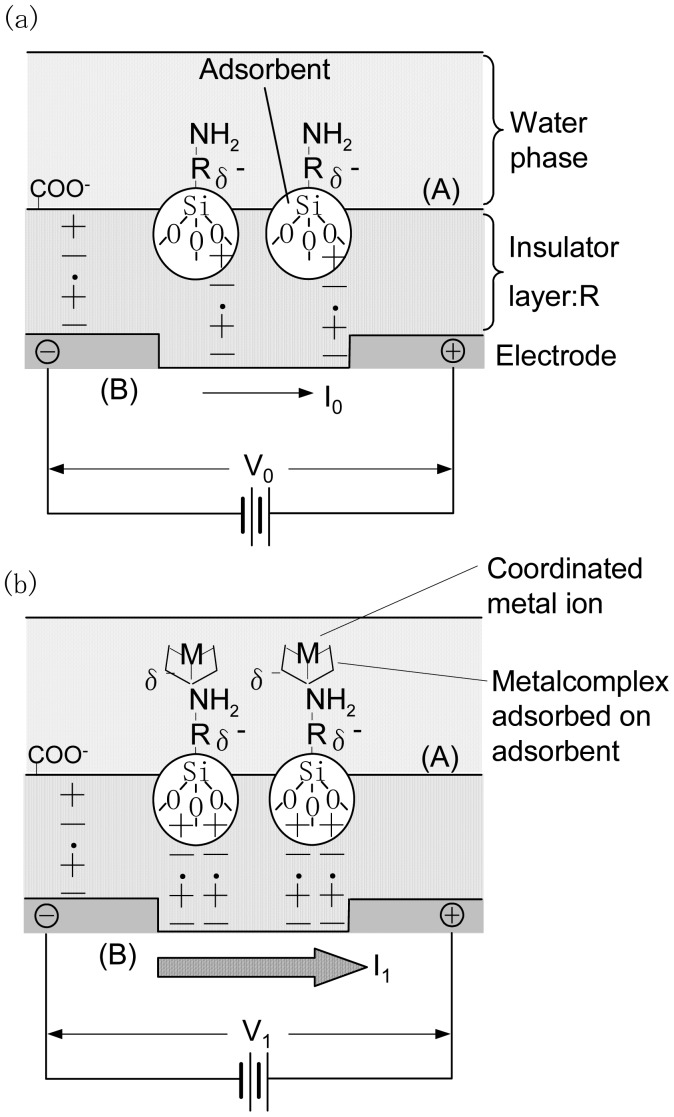
Schematic of an electrode chip and the detection mechanism of chemisorbed species.

Figure 1 shows a schematic of the structure and detection mechanism of a source-drain electrode coated with an insulation layer of submicron-order thicknessas obtained in this study. An ionic layer, like an electric double layer, forms at the surface of the solids and liquids [10]. When a solution containing metal ions with a concentration N is deposited onto the surface of an organic insulator with an adsorbent an electric double layer forms at the boundary surface(Figure 1(a)).Therefore, the insulator molecules become electrically polarized resulting in the formation of opposing charges on both the boundary surface(side (A) in Figure 1(a)) and the reverse side(side (B) in Figure 1(a)) of the insulator. This charge is detected by the source-drain electrode and is defined as the background voltage V_o_.

Figure 1(b) shows the detection mechanism of the adsorbed chelate molecule. When a solution containing metal ions of the same concentration N is deposited onto the surface of an insulator with an adsorbed chelate molecule (Figure 1(b), side A) coordination reaction between the specific metal ion and the chelate molecule occurs resulting in a charge at the reverse side of the insulator (Figure 1(b),side B). In this case, the relationship between the source-drain electrode current and the sensor voltage follows Ohm's law [10]. The detection voltage is defined as V_1_.

The adsorption ability of a chelate molecule on solid surface is obtained as follows:

(1) $$\text{Ion resonse}={\text{V}}_{1}/{\text{V}}_{\text{o}}$$

where V_1_is voltage detected using an electrode chip adsorbed chelate molecule while V_o_ is the detected voltage of a normal electrode chip.

### 2.2. Fabrication of an Electrode with an Adsorbed Chelate Molecule

**Figure 2.**
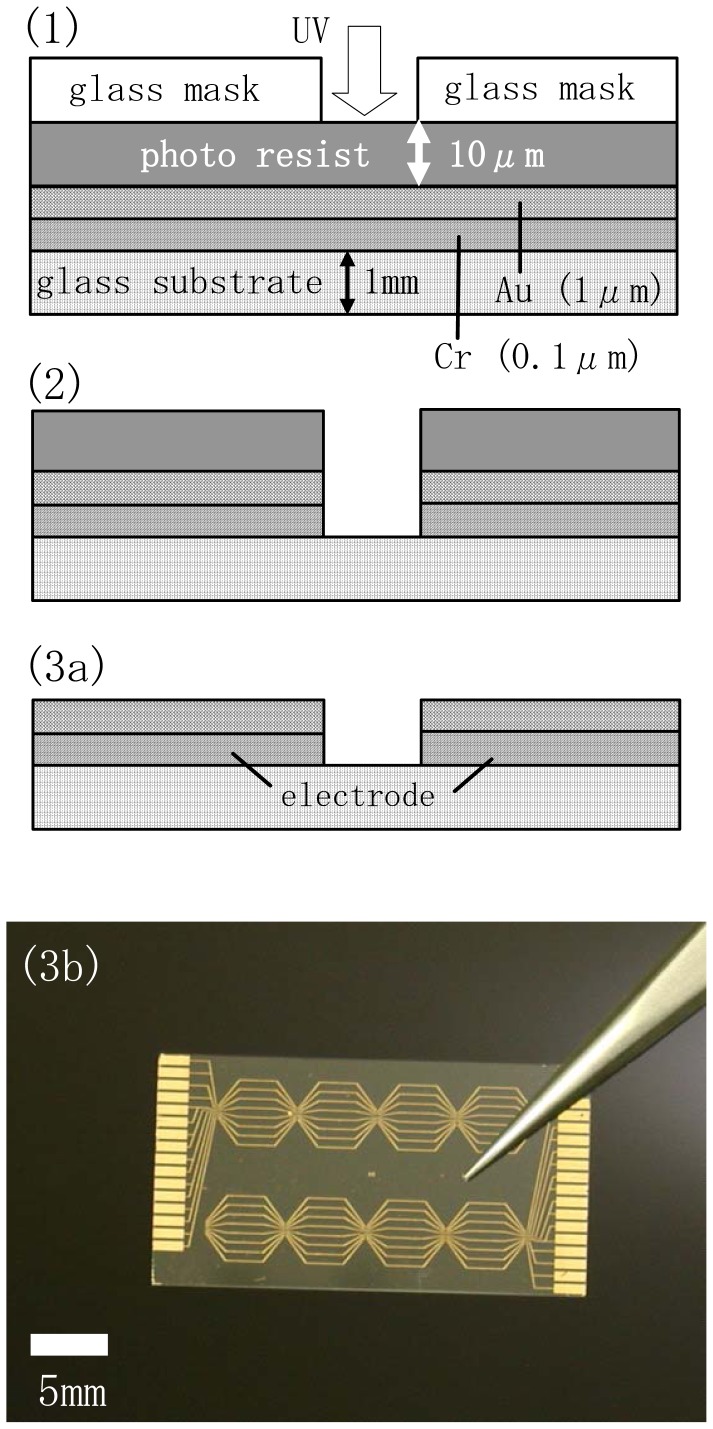
Fabrication steps for an electrode chip (**1**–**3a**).Photograph (**3b**) is an overview image of (**3a**).

Figure 2 shows the fabrication steps for the electrode chip as a part of the preparation of the source-drain electrodes coated with an insulation layer and with chelate molecules. A Cr adhesion layer (0.1 μm thick) and a Au layer (1 μm thick) were coated successively onto a glass substrate (20mm×30mm in size and 1 mm thick) using the sputter method. A positive-type photoresist was first spin-coated onto the Au surface and then patterned by irradiating the photoresist through a Cr-coated soda-lime glass mask with UV light (Figure 2(1)).After irradiation the exposed regions of the resist were removed using a 25% tetramethylammonium hydroxide (TMAH) solution (Figure 2(2)). The underlying Auregions were then chemically etched using a KI-I_2_ mixed solution (KI/I_2_=1/1g, concentration: 17 wt%) (Figure 2(2)). Patterning of the Crlayer was also carried out using a ceric ammonium nitrate solution (concentration: 5 wt%)for chemical etching(Figure 2(2)). The remaining photoresist layer was removed by washing extensively in acetone(Figure 2(3a)). An overview image of an electrode chip is shown in Figure 2(3b).Five pairs of electrodes were included in the hexagonal eight array units and were arranged on a chip with an area of 15×28 mm. One array unit was composed of two source- (or positive terminal) and five drain- (or negative terminal) electrodes arranged within an area of 5×4 mm. The length, width and inter-electrode spacing of eachelectrode were fixed at 3mm, 150μm and 50μm, respectively. The electrodes were connected by pads arranged at the edges of the chip, which in turn are connected to an amplification circuit in the sensor signal analyzer. The fabrication and signal detection mechanism of this sensor signal analyzer is described in our previous report[10].

**Figure 3.**
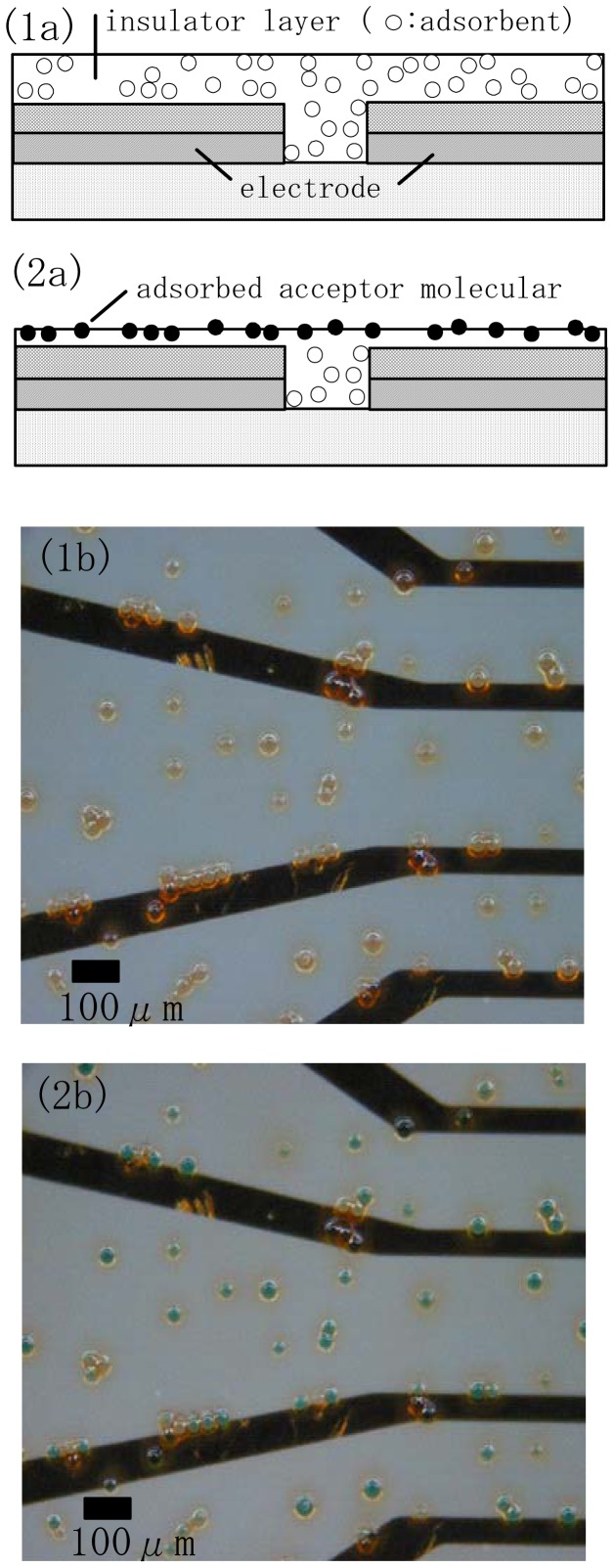
Fabrication steps for an electrode chip (**1a**–**2a**).Microscope images show an inter-electrode on a chip before (**1b**) and after (**2b**) wet-etching treatment.

An insulator layer was formed on the source-drain electrode by first spin-coating a novolac resin solution with SiO_2_ particles substituted with amino groups (Tosoh Co., 10μmφ, specific surface area: 200 m^2^/g) on the electrode and then drying at room temperature for 15 min(Figure 3(1a)). The insulator layer was then physically etched with a calcium carbonate particle solution using an ultrasonic cleaner. Chelate molecules dissolved in distilled water at a given concentration and volume were then deposited onto the insulator layer in the presence of an adsorbent (Figure 3(2a)). After adsorption treatment for 30min the electrode chip adsorbed chelate molecule was washed with distilled water to provide an electrode surface for evaluation.

Figure 3 shows an optical microscope image of the inter-electrode spacing before and after adsorption treatment. Here, copper phthalocyanine was used as a dye for staining. After the adsorption of the dye and washing with distillated water all the SiO_2_ particles were stained a green color (Figure 3(2b)), whereas the color was transparent before the adsorption of the dye (Figure 3(1b)).

### 2.3. Fabrication of a Wireless Sensor Screening System

**Figure 4.**
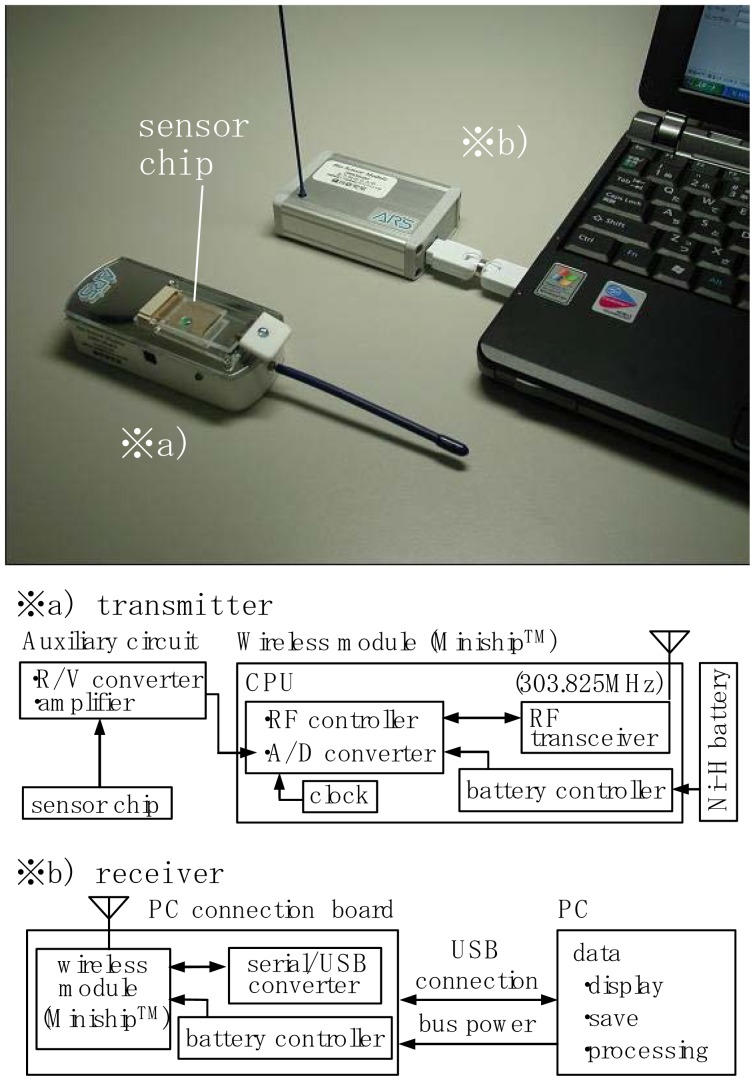
Wireless sensor signal analyzer system for the electrode chips. System flow of transmitter (**a**)and receiver (**b**).

Figure 4 shows a developed wireless sensor-screening system for the detection of signals from the electrode chip. Before treatment of the chelate molecules adsorbed on the electrodes, the electrode chip was attached to a transmitter(overall size: 24.5×20×90.5mm, weight: 70g, Figure 4(a)). When a solution containing a specific ion was spotted on the array the converted digital signals from the electrodes were transmitted to a receiver(Figure 4(b)) located some 5m from the point of detection.

The developed wireless system in a package module “Miniship^TM^” was incorporated into both the transmitter and the receiver (Figure 4(a,b)). The device, which has dimensions of 25×25×10 mm and a weight of 7g includes a PIC-type CPU. The power needed to drive this device (3.7V) is supplied by a Ni-H rechargeable battery fitted within the transmitter and/or attached via USB to a PC. The performance of the device has been reported previously [10,11].

The electrodes mounted on the electrode chip were connected to an R/V converter with an amplifier circuit. Each sensor signal is A/D converted by a CPU driven by a battery controller unit and a clock generator unit. The digital signal was transmitted by an RF transceiver (Figure 4(a)).A transmitted signal of 303.825MHz was received by the same device and was converted into a current signal by a serial/USB converter (Figure 4(b)). The current signal sent to the PC was then analyzed and processed using appropriate software.

### 2.4. Evaluation of the Adsorbed Chelate Molecules Using an Electrode Chip

The chelate molecules used for the adsorption experiment are given in Table 1.*O*,*O*′-bis(2-Aminoethyl)ethyleneglycol-*N*,*N*,*N*′,*N*′-tetraacetic acid (GEDTA) as a Ca^2+^chelator[CA(G)], *O*,*O*&prime-bis(2-aminophenyl)ethyleneglycol-*N*,*N*,*N*′,*N*′-tetraacetic acid, tetrapotassium salt, hydrate (BAPTA) as a Ca^2+^ chelator [CA(B)], 3-[3-(2,4-dimethylphenylcarbamoyl)-2-hydroxynaphthalene-1-yl-azo]-4-hydroxybenzenesulfonic acid, sodium salt (XB-1) as a Mg^2+^chelator (MG), 2-nitroso-5-[*N*-*n*-propyl-*N*-(3-sulfopropyl)amino]phenol (nitroso-PSAP) as an Fe^2+^ chelator [FE(N)], 4,7-diphenyl-1,10-phenanthrolinedisulfonic acid, disodium salt (BPDSA) as an Fe^2+^ chelator [FE(B)], 2,9-dimethyl-4,7-diphenyl-1,10-phenanthrolinedisulfonic acid, disodium salt (BCIDSA) as a Cu^2+^ chelator [CU(I)] and 5,10,15,20-tetrakis(*N*-methylpyridinium-4-yl)-21*H*,23*H*-porphine,tetrakis(*p*-toluenesulfonate) (TMPyP) as a Cu^2+^ chelator [CU(II)] were used for adsorption onto electrode chips. These commercial chemicals were provided by Dojindo Laboratories (Kumamoto, Japan).Solutions of CaCl_2_, MgCl_2_, FeCl_2_ and CuCl_2_ were used as guest molecules for adsorption onto the chelators. These commercial chemicals were provided by Wako Pure Chemical Industries, Ltd (Osaka, Japan).The combination of guest cations and chelators as host molecules for electrode detection are also summarized in Table 1.

Before an evaluation of the host-guest reaction on the electrode, a chelate dissolved in 200μL distilled water (concentration of 10mg/mL) was prepared and added drop wise to the surface of an insulator layer on the electrode chip. After adsorption treatment for 30min the electrode chip was washed with distilled water and used for an evaluation of the host-guest reaction.

Two kinds of cation solution were prepared for an evaluation of the adsorbed chelate molecules on the electrode chips: (a) only one type of cation dissolved in distilled water at three given concentrations of 1×10^−2^mol·L^−1^, 1×10^−4^mol·L^−1^ and 1×10^−6^mol·L^−1^ (solution-free buffer) and (b) cations dissolved in a model buffer solution at three given concentrations of 1×10^−2^mol·L^−1^, 1×10^−4^mol·L^−1^ and 1×10^−6^mol·L^−1^ (supplemental buffer solution). A model buffer solution was prepared using Na, K, Ca and Mg chloride (Na^+^ : K^+^: Ca^2+^ : Mg^2+^ = 95 : 3.3 : 1.7 : 1.0 mol for a total concentration of 1×10^−2^mol·L^−1^) in distilled water (“DW”). The model buffer solution was added to the solution-free buffer of 1×10^−2^mol·L^−1^ with a volume ratio of 1:1 (“standard solution”). The supplemental buffer solution was prepared by diluting the standard solution to 1/100 and 1/10,000. For Ca^2+^ or Mg^2+^ ion measurements a standard solution was prepared without the addition of CaCl_2_ or MgCl_2_, respectively.

Experiments were carried out as follows:

- PC software for the wireless sensor-screening system was started and an electrode voltage was detected.
- After a period of 10 s to stabilize the device a cation solution of 20 μL was deposited onto the electrode chip for 180 s.
- After the measurement the cation solution was removed.
- Steps (i) to (iii) were repeated for the three solution-free buffer samples and the three supplemental buffer solution samples.

We have previously reported that the present sensor screening system rapidly detects voltage (within 100 ms) after the deposition of a sample solution and each electrode's profile was mostly stable [10]. Measurement of this sensor has been also performed. Therefore, the detected voltage taken between time intervals of 120 and 180s were averaged and an electrode voltage was obtained. Further more, the ion selectivity parameter, as defined by Equation(1), was also obtained.

### 2.5. Molecular Orbital Calculations for the Chelate Molecules

Molecular orbital (MO) calculations for the chelate molecules were carried out with commercial software (MOPAC Vr.3, Fujitsu Co. Tokyo, Japan) using the PM3 method [12]. The energy level of the highest occupied MO (HOMO) and the lowest unoccupied MO (LUMO) were obtained by this calculation method. Furthermore, the HOMO and LUMO level of the model structure, as a surface of adsorbent, was also determined. Details are referred to in the discussion section.

## 3. Results

### 3.1. Detection of Strong Interactions between the Chelate Molecules Adsorbed on the Electrode Chips and the Cations

**Figure 5.**
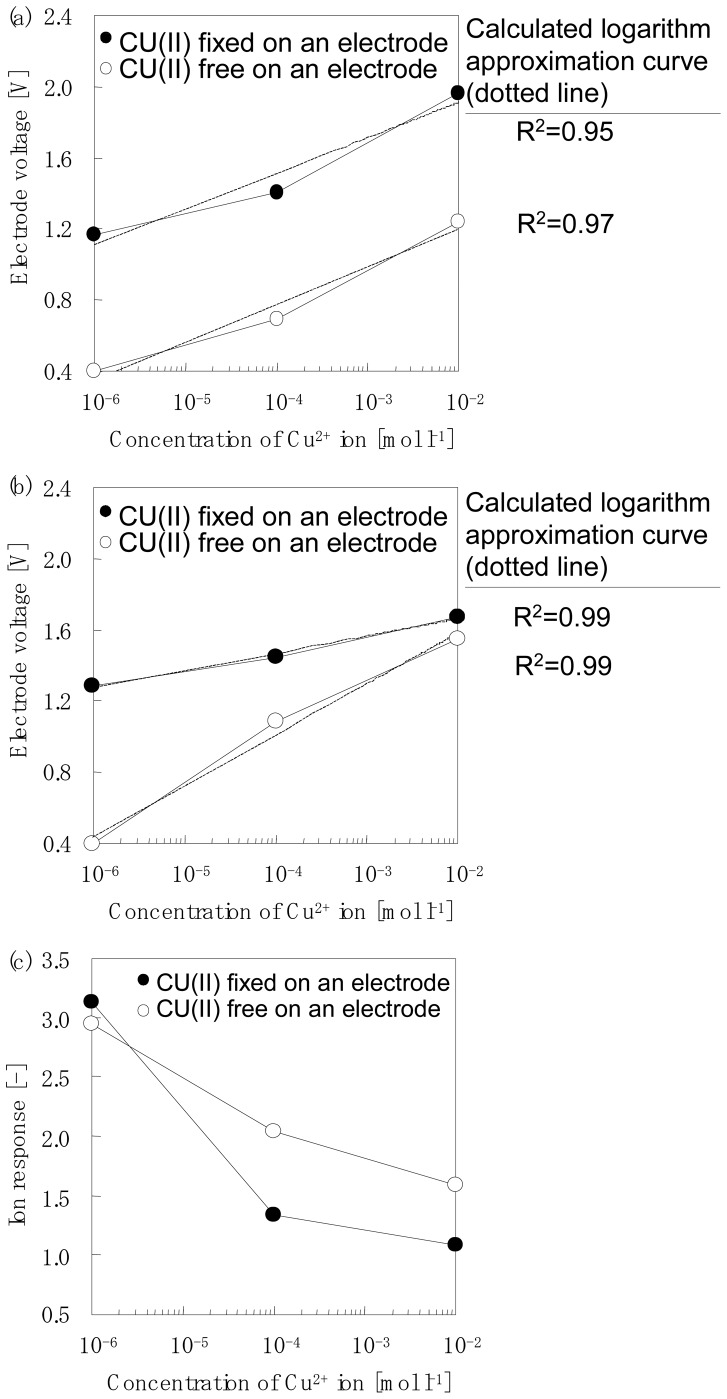
Typical detection voltage of a type I host-guest reaction; group of electrodes that show a positive response. Detection of Cu^2+^ by CU(II) adsorbed on an electrode surface are shown in Figure 5(a, b), respectively (measurement sample: only one type of cation dissolved in distilled water (Figure 5(a)), cation dissolved in buffer solution (Figure 5(b)) Example of a typical ion response of a type I host-guest reaction is also shown in Figure 5(c).

Detection voltage details for the electrodes with the adsorbed chelate molecules are summarized in Table 2. Group1 contains four kinds of chelate molecules; CU(II), MG, FE(N) and FE(B), respectively. Figures 5 shows an example of the typical detection voltage of the host-guest interaction between the chelate molecules (Group1) adsorbed on the sensor electrodes and the cations. Figure 5(a) shows the detection of Cu^2+^ in pure water by CU(II) adsorbed on an electrode surface. The white plot of the solid line shows the relationship between the concentration of the Cu^2+^ ion and the voltage of the electrode without adsorption of the CU(II) chelate. The electrode voltage increased with an increase in the Cu^2+^ concentration giving values of 0.4 V at 1×10^−6^ mol·L^−1^, 0.69 V at 1×10^−4^ mol·L^−1^ and 1.24 V at 1×10^−2^ mol·L^−1^, respectively. The dotted line shows the logarithm is an approximate straight line. A coefficient of correlation R^2^ value of 0.97 was obtained.

The black plot of the solid line shows the relationship between the concentration of Cu^2+^ ions and the voltage of the electrode with adsorbed CU(II) chelate. After the adsorption of the CU(II) chelate the detected electrode voltages increased to 0.7V and the voltage increased with an increase in Cu^2+^ concentration. The obtained values were 1.17 V at 1× 10^−6^ mol·L^−1^, 1.41 V at 1× 10^−4^ mol·L^−1^ and 1.96 V at 1× 10^−2^ mol·L^−1^, respectively. A coefficient of correlation value of 0.95 was obtained.

Figure 5(b) shows the detection of Cu^2+^ in the presence of a buffer solution by CU(II) adsorbed onto an electrode surface. The white plot of the solid line shows the relationship between the concentration of the Cu^2+^ ion contained in the buffer solution and the voltage of the electrode without the adsorption of the CU(II) chelate.

The electrode voltage increased with an increase in the Cu^2+^ concentration and values of 0.41 V at 1×10^−6^ mol·L^−1^, 1.09 V at 1×10^−4^ mol·L^−1^ and 1.55 V at 1×10^−2^ mol·L^−1^were obtained, respectively. The dotted line shows the logarithm is an approximate straight line. A coefficient of correlation R^2^ value of 0.99 was obtained.

The black plot of the solid line shows the relationship between the concentration of the Cu^2+^ ions contained in a buffer solution and the voltage of the electrode with adsorbed CU(II) chelate. After the adsorption of the CU(II) chelate the electrode voltage increased with an increase in the Cu^2+^ concentration giving values of 1.29 V at 1×10^−6^ mol·L^−1^, 1.45 V at 1×10^−4^ mol·L^−1^ and 1.68 V at 1×10^−2^ mol·L^−1^, respectively. Electrode detection was hindered by the buffer solution and the voltage increased by 0.13V at 1×10^−2^ mol·L^−1^ of Cu^2+^ in a buffer solution after the CU(II) chelate was absorbed onto the electrode. A coefficient of correlation value of 0.99 was obtained.

Figure 5(c) shows an example of a typical ion response to a type I host-guest reaction. The detection of Cu^2+^by the CU(II) chelate that was adsorbed on an electrode surface is shown in Figure 5(c).The white plot of the solid line shows a relationship between the concentration of the Cu^2+^ ion in solution and the ion responsibility of the electrode adsorbed CU(II) chelate. The ion response decreased with an increase in Cu^2+^ concentration with values of 2.93 at 1×10^−6^ mol·L^−1^, 2.04 at 1×10^−4^ mol·L^−1^ and 1.58 at 1×10^−2^ mol·L^−1^, respectively. The black plot of the solid line shows the relationship between the concentration of the Cu^2+^ ion contained in a buffer solution and the ion response of the electrode adsorbed CU(II) chelate. Ion response decreased with an increase in theCu^2+^ concentration with values of 3.15 at 1×10^−6^ mol·L^−1^, 1.33 at 1×10^−4^ mol·L^−1^ and 1.08 at 1×10^−2^ mol·L^−1^, respectively. The ion response decreased to 35% at a concentration of more than 1×10^−4^ mol·L^−1^ of Cu^2+^ in a buffer solution, while the ion response had no influence at 1×10^−6^ mol·L^−1^ Cu^2+^ in a buffer solution and/or a buffer free solution.

### 3.2. Detection of Weak Interactions between Chelates Adsorbed on Electrode Chips and Cations

**Figure 6.**
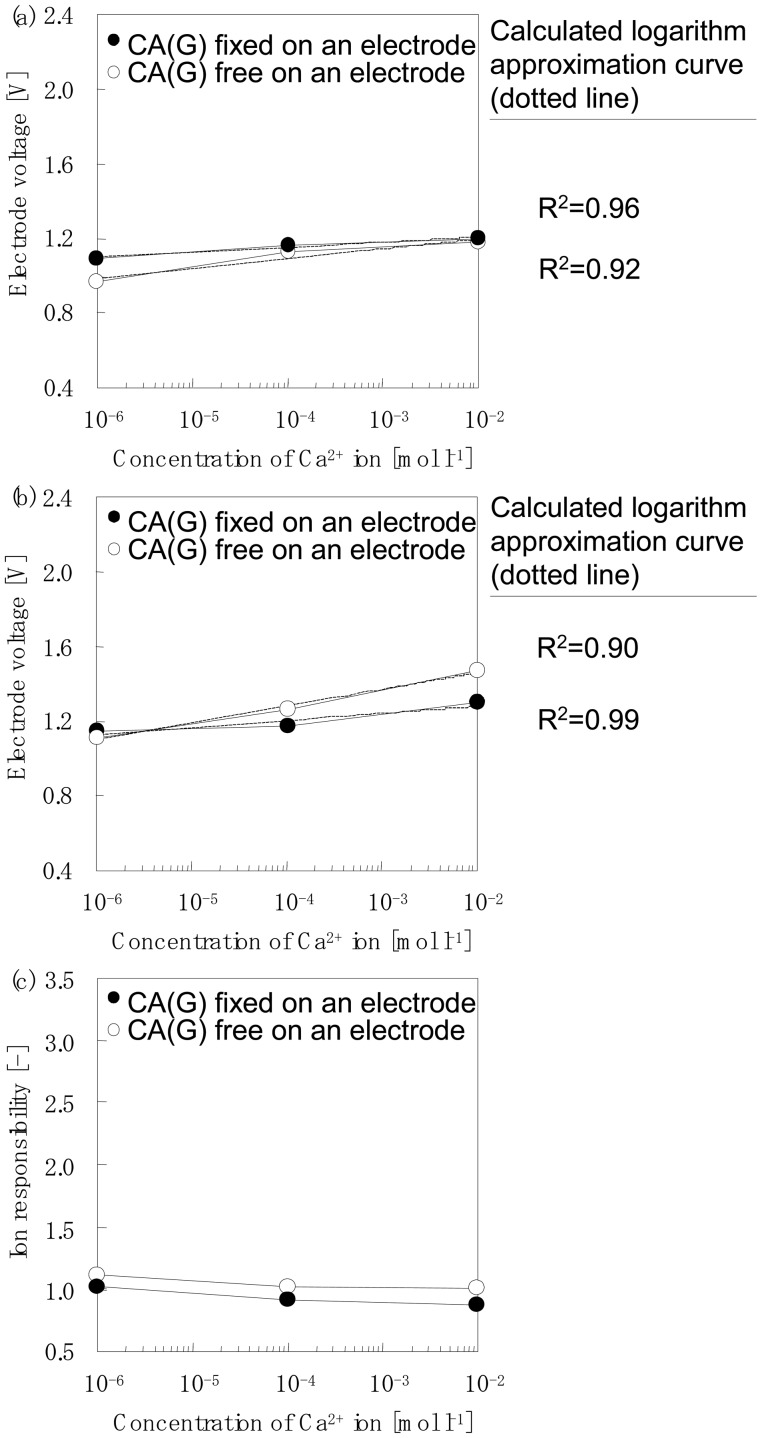
Example of the typical detection voltage of a type II host-guest reaction; a group of electrodes that show negative response. Detection of Ca^2+^by CA(G) adsorbed on an electrode surface are shown in Figure 6(a, b). Measurement sample: only one type of cation dissolved in distilled water (Figure 6(a)); cation dissolved in buffer solution (Figure 6(b)). Example of a typical ion response of a type II host-guest reaction is also shown in Figure 6(c).

The detection voltage details from the electrodes containing the adsorbed chelate moleculesare summarized in Table 3. Group 2 contains three kinds of chelate molecules: CU(I), CA(B) and CA(G), respectively. Figures 6 show an example of the typical detection voltage of the host-guest reaction between the chelate molecules (Group2) that are adsorbed on the electrode and the cations. Figure 6a shows the detection of Ca^2+^ in pure water by CA(G) adsorbed onto an electrode surface. The white plot of the solid line shows the relationship between the concentration of the Ca^2+^ ions and the voltage of the electrode without an adsorbed CA(G) chelate. The electrode voltage increased slightly with an increase in the Ca^2+^ concentration with values of 0.97 V at 1×10^−6^ mol·L^−1^, 1.13 V at 1×10^−4^ mol·L^−1^ and 1.18 V at 1×10^−2^ mol·L^−1^, respectively. The dotted line is a logarithm of an approximate straight line.A coefficient of correlation R^2^ value of 0.92 was obtained.

The black plot of the solid line shows the relationship between the concentration of the Ca^2+^ ion and the voltage of the electrode adsorbed CA(G) chelate. After the adsorption of the CA(G) chelate, the detection voltage hardly changed and gave values of 1.09 V at 1×10^−6^ mol·L^−1^, 1.17 V at 1×10^−4^ mol·L^−1^ and 1.20 V at 1×10^−2^ mol·L^−1^, respectively. A coefficient of correlation value of 0.96 was obtained.

Figure 6(b) shows the detection of Ca^2+^ in the presence of a buffer solution by the CA(G) adsorbed onto an electrode surface. The white plot of the solid line shows the relationship between the concentration of the Ca^2+^ ions contained in a buffer solution and the voltage of the electrodes without the CA(G) chelate. The electrode voltage hardly changed and gave values of 1.11 V at 1×10^−6^ mol·L^−1^, 1.27 V at 1×10^−4^ mol·L^−1^ and 1.47 V at 1×10^−2^ mol·L^−1^, respectively.

The black plot of the solid line shows the relationship between the concentration of the Ca^2+^ ions contained in a buffer solution and the voltage of the electrode adsorbed CA(G) chelate. After the adsorption of the CA(G) chelate the electrode voltage increased slightly with an increase in the Ca^2+^ concentration and gave values of 1.15 V at 1×10^−6^ mol·L^−1^, 1.18 V at 1×10^−4^ mol·L^−1^ and 1.30 V at 1×10^−2^ mol·L^−1^, respectively. A coefficient of correlation value of 0.99 was obtained.

Figure 6(c) shows an example of the typical ion response of a type II host-guest reaction. The detection of Ca^2+^ by the CA(G) chelate that was adsorbed on an electrode surface is shown in Figure 6(c). In this case, the absorption of the CA(G) chelate made no difference.

### 3.3. Molecular Orbital Calculations for theChelate Molecules

To predict the interaction between the chelate molecules and the adsorbent the electronic orbitals of the chelate molecules and the surface model structures of the adsorbent was determined by MO calculations. The HOMO- and LUMO-values of each molecule are summarized in Table 4. Seven kinds of chelates, which were examined during the electrode evaluations, were subjected to calculations. The HOMO- and LUMO-values of the surface molecular structure are also summarized in Table 4.The SiO_2_ surface model was assembled using five SiO_2_clusters, which were minimized units. The clusters were further connected to an aminoalkyl group and a NH_2_-R-(SiO_2_)_n_(R=–C_2_H_4_–NH–C_2_H_4_–, n=5) cluster was constructed. MO calculations for the assembled cluster model were also carried out and a calculated HOMO' value of−8.890eVwas obtained.

In accordance with frontier orbital theory [13],a chemical reaction between two molecules occurs easily when the difference between the HOMO- and LUMO-level is small. An amino group is present on the model surface structure as well as an NH_2_-R-(SiO_2_)_n_ cluster. The nitrogen atom in the amino group has a lone pair of electrons that can be donated to another molecular orbital. The lowest unoccupied MO (LUMO) of the chelate molecules can accept a lone-pair electron. Therefore, the difference between the HOMO'-level of the NH_2_-R-(SiO_2_)_n_ cluster and the LUMO-level of the chelate molecules were calculated. The difference is described as Δ(LUMO-HOMO') and is summarized in Table 4. Various values for Δ(LUMO-HOMO') were obtained. The CA(B) chelate gave the maximum value of 10.302 eV and the CU(II) chelate gave the lowest value of 5.472 eV, respectively.

**Figure 7.**
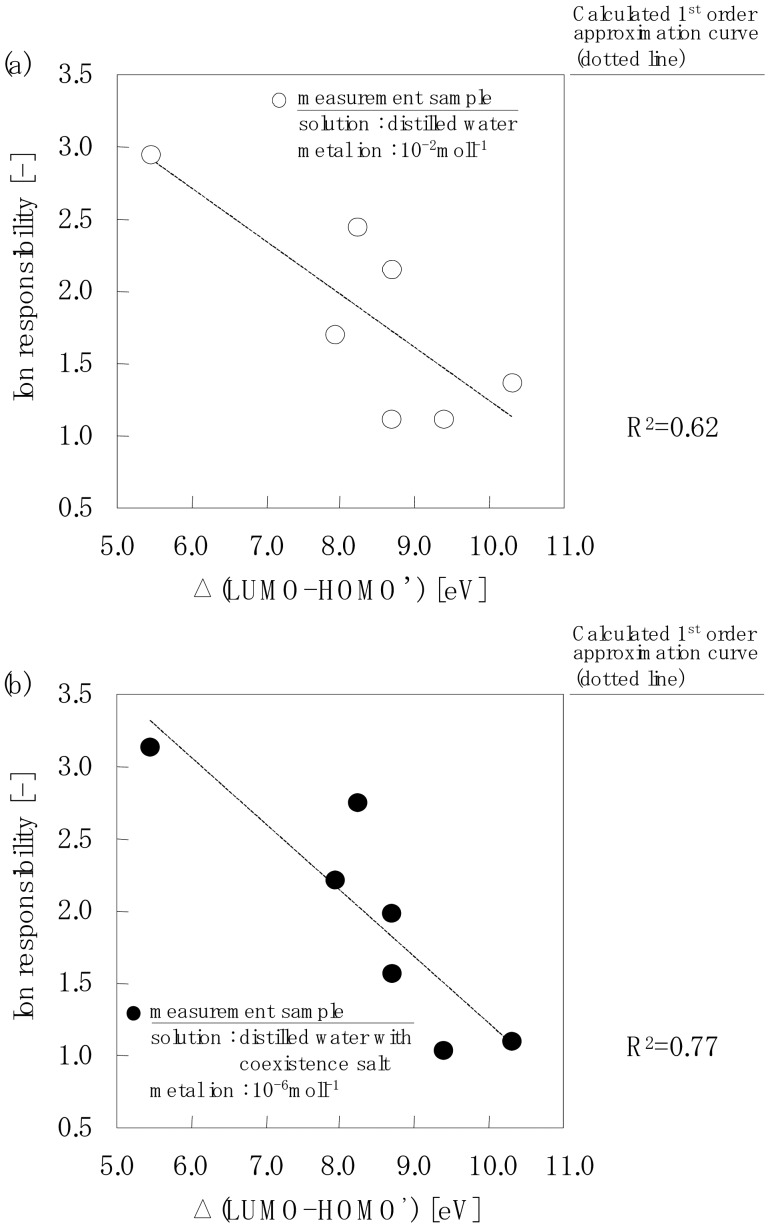
Relationships between the actual measured ion response value using an electrode chip and the calculated Δ(LUMO-HOMO')value.

Figure 7 shows the relationships between the actual measurement values of the ion response using an electrode chip and the calculated Δ(LUMO-HOMO') value. Figure 7(a) shows the ion response of all the chelate molecules when the measurement sample solution contained 10^−2^mol·L^−1^ metal ions. The actual measurement value of the ion response decreased with an increase in the Δ(LUMO-HOMO') value; however, the 1st order approximation curve gave 0.62.

Figure 7(b) shows the ion response of each of the chelate molecules when the measurement sample solution contained 10^−6^mol·L^−1^ metal ions. The actual measurement value of the ion response decreased with an increase in the Δ(LUMO-HOMO') value with a 1st order approximation curve of 0.77.

## 4. Discussion

The selective detection of inorganic ions using an electrode has been extensively reported. In one example the electrochemical response of a phenanthroline derivatives electrochemically grafted to glassy carbon (GC) for the determination of the Cu(II) ions was examined[14]. GC-based electrodes were electrochemically modified by layers of phenanthroline derivatives such as poly-5-nitro-1,10-phenanthroline (poly-5NP), *etc*. The applicability of poly-5NP-functionalized carbon surfaces for the determination of Cu(II) ions was demonstrated by formation of complexes between GC-grafted poly-5NP derivative layers and Cu(II) ions. Electrochemical determination of Cu(II) ions by a 4-formylphenylboronic acid modified gold electrode was also examined [15]. A self-assembled monolayer modified by 4-formylphenylboronic acid was formed on the gold electrode, which was applied for the determination of trace concentrations of Cu(II).Other examples have also been reported such as the measurement Mg(II) [16], Ca(II) [17,18], Fe(III) [19], Zn(II) [20,21], respectively. These previous studies have been shown high performance for selectivity of specific ion. Here, a specific cation was coordinated with a complex compound that was immobilized on an indicating electrode and an electromotive force between the indicating electrode and the reference electrode was detected. The advantage of an ion electrode is wide concentration range for the detection and is high selectivity for a specific ion.

In contrast, the electrode chip described in this study measures the amount of polarization, which arises from the polymer-based insulator layer containing inorganic adsorbent particles using an electrode pair. A chelate is chemically adsorbed onto the surface of the SiO_2_ particles and is held there by the surface of the insulation layer.

The amount of supported complex molecule depends on the surface area of SiO_2_ particles exposed on the surface of an insulate layer. When the ion concentration in solution is lower (10^−6^mol·L^−1^), the complexation reaction progresses quantitatively on the SiO_2_ surface. Therefore the ion selectivity of the sensor becomes high. On the other hand, complexation reactions reach saturation at higher ion concentrations (10^−2^mol·L^−1^) in solution. As a result, ion selectivity is decreased. When complex compounds of Type I is used, this tendency is more remarkable than that of the ion electrode.

Furthermore, the reason is discussed why ion selectivity was different between complex compounds of Type I and Type II group. The adsorption of the chelate could be evaluated indirectly by this measurement based on the response of this chemical adsorption species and the specific ion. The ion response defined by Equation (1) is the ratio of the two states of the electrode voltage; one is an electrode adsorbed by a chelate compound and the other is a normal chelate-free electrode. In other words, when the ion response values increase more than 1.0, the amount of chelate adsorption on the surface of the electrode is expected to increase. For example, Figure 5(c) clearly shows that some chelates are strongly adsorbed onto the surface of the SiO_2_ particles modified by the amino-group. On the other hand, Figure 6(c) shows that some chelates are weakly adsorbed onto the same adsorbent surface.

A lone pair electron is present on the nitrogen atom in the amino-group. This molecular orbital is the HOMO' as determined by MO calculations and this is shown in Table 4, and it does not contradict theory. In accordance with frontier orbital theory, the molecular orbital on the chelateside corresponds to the LUMO and it can interact with the HOMO' on the surface of the SiO_2_ particles. Figure 7 shows that the ion response, which is the relative rate of the amount of chelate adsorption (experimental value) and Δ(LUMO-HOMO') (theoretical value), which is a parameter of the interaction, shows high correlation.

**Figure 8.**
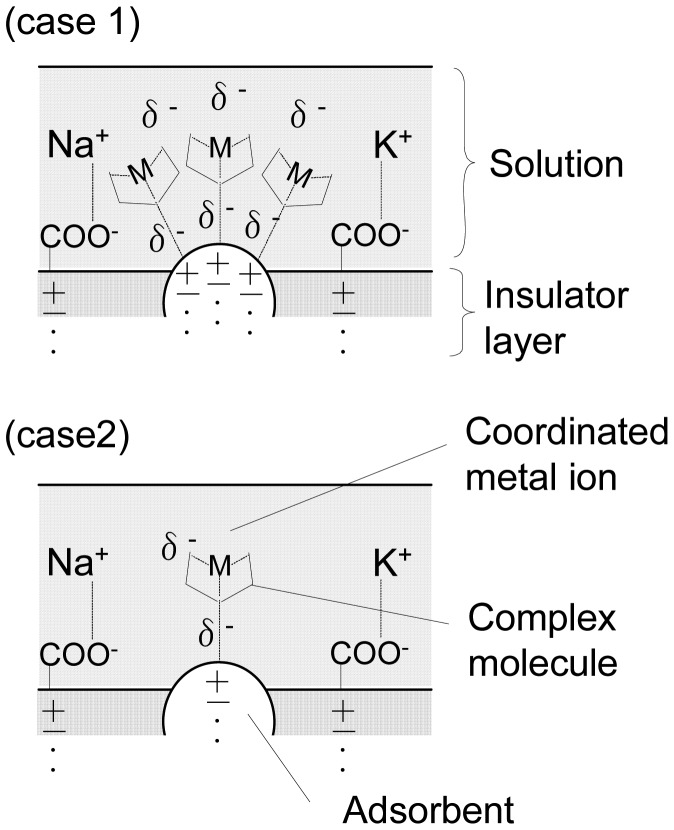
Schematic of the difference in adsorption detection ability.

Figure 8 shows a schematic model of the basis of these results. Therefore, we estimate that chelates that can interact with the HOMO' on an amino group are absorbed onto the surface of SiO_2_(Figure 8 (case 1)). These chemisorbed species react to a specific ion and an increase in the electrode voltage is observed as the chelation reaction changes. On the other hand, we expect that chelates that hardly interact with the HOMO' of an amino group do not absorb well onto the surface of SiO_2_(Figure 8 (case 2)). The electrode surface did not react to a specific ion and this is because of a change in the low electrode voltage. This study clarified that difference in adsorption on the electrode of complex molecule is influenced for ion detection.

## 5. Conclusions

A chip-mounted microelectrode coated with an insulator layer and adsorbent SiO_2_ particles modified by an amino group to adsorb various chelate molecules was investigated with respect to its chelation reaction detection properties. These properties change upon an interaction with specific cations and their electronic states were investigated. This study clarified the following three points:

1. In accordance with the electrode evaluation results, groups exist wherein a chelate and a specific cation react on the surface of SiO_2_ particles modified by an amino-group. On the other hand, groups exist wherein chelate molecules and specific cations exhibit low reactivity on the same adsorbent surface.
2. In accordance with the results of molecular orbital calculations, a lone pair electron was located on the nitrogen atom in the amino group on the surface of the SiO_2_ particles. This molecular orbital was identified as being the HOMO'. On the other hand, the molecular orbital on the chelate compound side was predicted to be the LUMO by frontier orbital theory, which possibly interacts with the HOMO' on the surface of the SiO_2_ particles. Ion response, which is the relative rate of the amount of chelate adsorption (experimental value) and Δ(LUMO-HOMO') (theoretical value),as an interaction parameter showed high correlation.
3. Based on the above two results, the chelate compounds that can interact with the LUMO of amino groups absorb onto the surface of the SiO_2_. These chemisorbed species react with specific ions and an increase in electrode voltage is observed as the chelation reaction changes. On the other hand, the chelate compounds that hardly interact with the HOMO' of the amino group hardly absorbs onto the surface of SiO_2_. The electrode surface does not react to specific ions and we conclude that this is because of a change in the low sensor voltage.

We thus found that the design of the chemical structure of the surface of an adsorbent on an insulation layer fabricated on electrode pairsis an important factor. Optimization of the host molecule, *i.e.*, chelate compounds and/or antibodies and adsorbents on the electrode surface is important for immobilization. This is required for repeatable electrode-measurements.

## Figures and Tables

**Table 1.** Combination of donor- and acceptor-chemicals.

**Acceptor chemicals** (1) **fixed onto a sensor surface**					**Donor chemicals in solution**
**Abr.**	**Trade (1) name**	**Molecular formula**	**Functions**	**Molecular structure**	
CA(G)	GEDTA	C_14_H_24_N_2_O_10_	Ca^2+^chelator	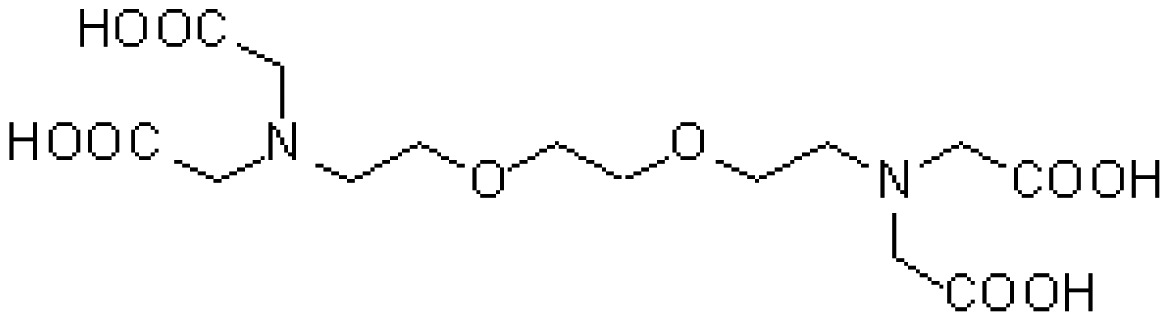	CaCl_2_
CA(B)	BAPTA	C_22_H_20_K_4_N_2_O_10_	Ca^2+^ chelator	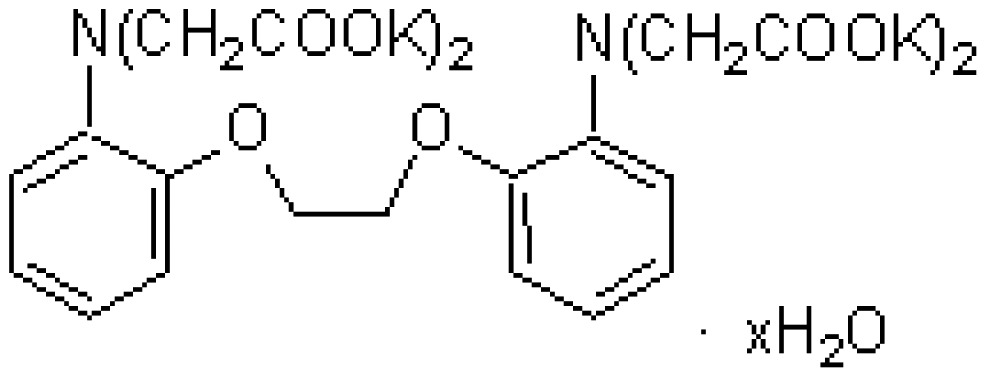	CaCl_2_
MG	XB-1	C_25_H_20_N_3_NaO_6_S	Mg^2+^chelator	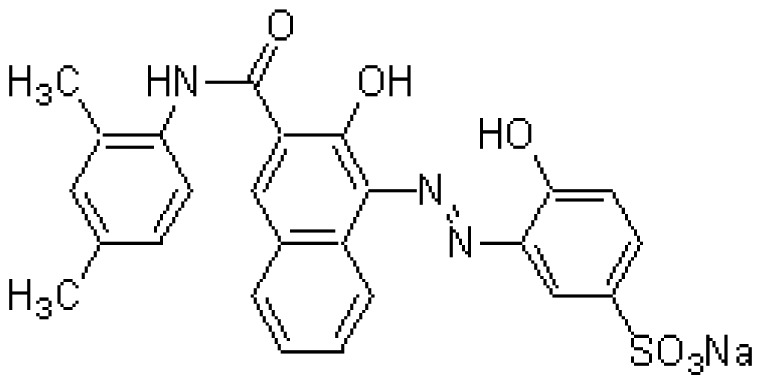	MgCl_2_
FE(N)	Nitroso-PSAP	C_12_H_18_N_2_O_5_S	Fe^2+^chelator	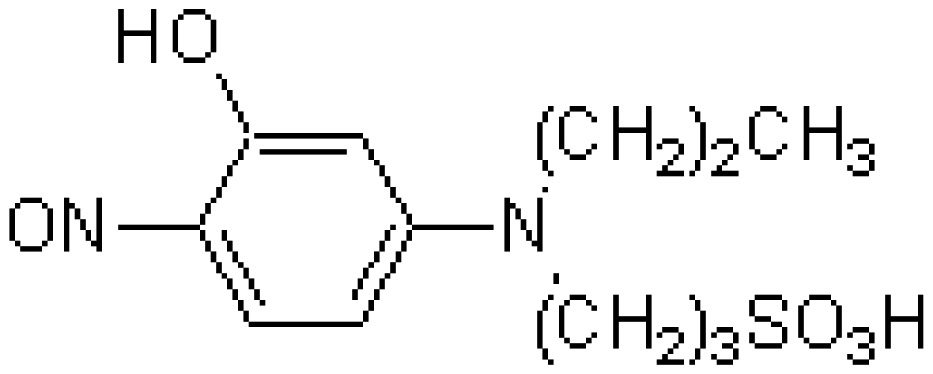	FeCl_2_
FE(B)	BPDSA	C_24_H_14_N_2_Na_2_O_6_S_2_	Fe^2+^chelator	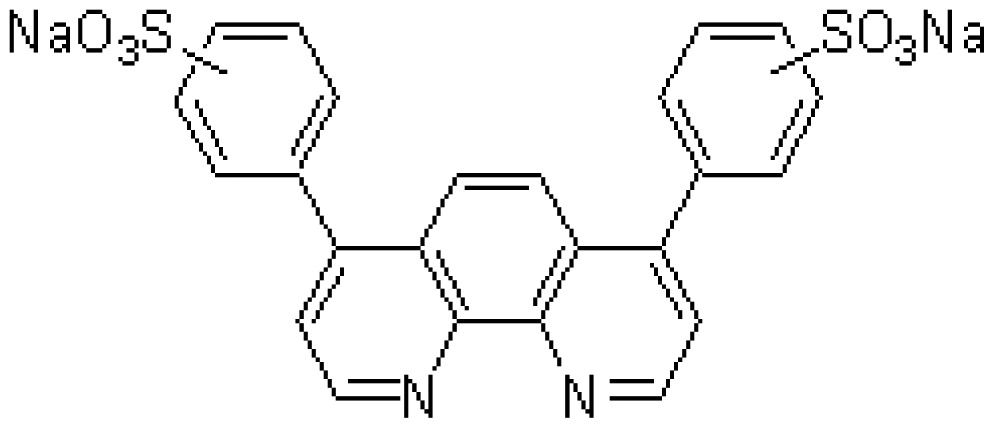	FeCl_2_
CU(I)	BCIDSA	C_26_H_18_N_2_Na_2_O_6_S_2_	Cu^2+^chelator	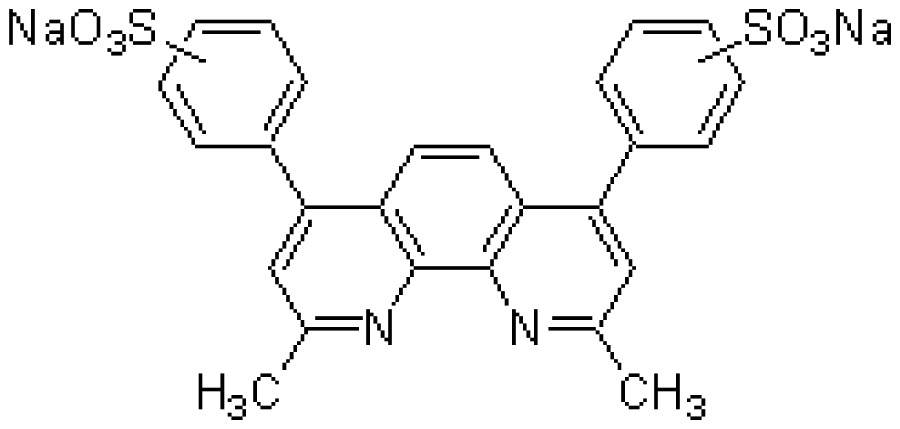	CuCl_2_
CU(II)	TMPyP	C_72_H_66_N_8_O_12_S_4_	Cu^2+^chelator	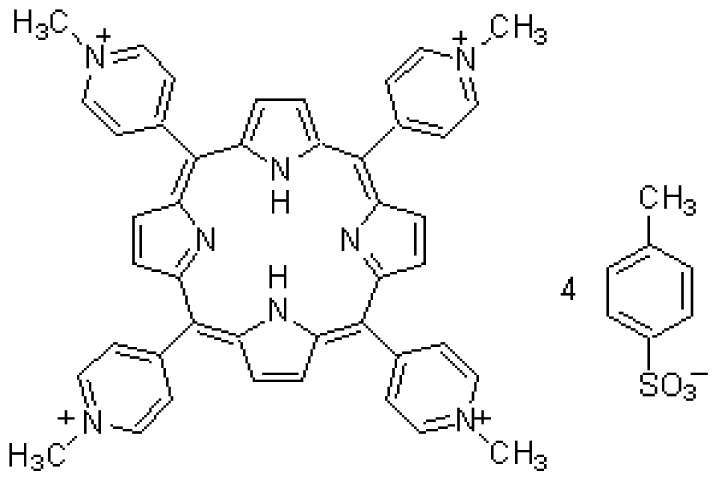	CuCl_2_

(1) Doujin Chemical Co.

**Table 2.** Detected electrode voltages from host-guest reactions (Type I).

(1) Detection of Cu^2+^ by CU(II) fixed on an electrode.				

**Measured substance**	**Concentration of substance[mol·L^−1^]**	**Electrode voltage [V]**		**Ion response**

		**State of an electrode surface**		

		**CU(II) free**	**Fixed CU(II)**	

Cu^2+^	1.00×10^−2^	1.24	1.96	1.58
	1.00×10^−4^	0.69	1.41	2.04
	1.00×10^−6^	0.40	1.17	2.93

Cu^2+^+BS	1.00×10^−2^	1.55	1.68	1.08
	1.00×10^−4^	1.09	1.45	1.33
	1.00×10^−6^	0.41	1.29	3.15

(2) Detection of Mg^2+^ by MG fixed on an electrode.				

**Measured substance**	**Concentration of substance [mol·L^−1^]**	**Electrode voltage [V]**		**Ion response**

		**State of an electrode surface**		

		**MG free**	**Fixed MG**	

Mg^2+^	1.00×10^−2^	0.77	1.55	2.01
	1.00×10^−4^	0.62	1.41	2.27
	1.00×10^−6^	0.52	1.27	2.44

Mg^2+^+BS	1.00×10^−2^	1.30	1.51	1.16
	1.00×10^−4^	0.70	1.43	2.04
	1.00×10^−6^	0.51	1.41	2.76

(3) Detection of Fe^2+^ by FE(N) fixed on an electrode.				

**Measured substance**	**Concentration of substance [mol·L^−1^]**	**Electrode voltage [V]**		**Ion response**

		**State of an electrode surface**		

		**FE(N) free**	**Fixed FE(N)**	

Fe^2+^	1.00×10^−2^	2.00	2.34	1.17
	1.00×10^−4^	0.94	1.45	1.54
	1.00×10^−6^	0.58	0.99	1.71

Fe^2+^+BS	1.00×10^−2^	1.58	2.21	1.40
	1.00×10^−4^	0.93	1.30	1.40
	1.00×10^−6^	0.50	1.11	2.22

(4) Detection of Fe^2+^ by FE(B) fixed on an electrode.				

**Measured substance**	**Concentration of substance [mol·L^−1^]**	**Electrode voltage [V]**		**Ion response**

		**State of an electrode surface**		

		**FE(B) free**	**Fixed FE(B)**	

Fe^2+^	1.00×10^−2^	2.00	2.27	1.14
	1.00×10^−4^	0.94	1.38	1.47
	1.00×10^−6^	0.58	1.25	2.16

Fe^2+^+BS	1.00×10^−2^	1.58	1.85	1.17
	1.00×10^−4^	0.93	1.14	1.23
	1.00×10^−6^	0.50	1.00	2.00

**Table 3.** Detected electrode voltage by host-guest reaction (Type II).

(1) Detection of Cu^2+^ by CU(I) fixed on an electrode.				

**Measured substance**	**Concentration of substance [molL^−1^]**	**Electrode voltage [V]**		**Ion response**

		**State of an electrode surface**		

		**CU(I) free**	**Fixed CU(I)**	

Cu^2+^	1.00×10^−2^	1.24	1.51	1.22
	1.00×10^−4^	0.69	0.94	1.36
	1.00×10^−6^	0.40	0.44	1.10

Cu^2+^+BS	1.00×10^−2^	1.55	1.05	0.97
	1.00×10^−4^	1.09	1.11	1.02
	1.00×10^−6^	0.41	0.65	1.59

(2) Detection of Ca^2+^ by CA(B) fixed on an electrode.				

**Measured substance**	**Concentration of substance [molL^−1^]**	**Electrode voltage [V]**		**Ion response**

		**State of an electrode surface**		

		**CA(B) free**	**Fixed CA(B)**	

Ca^2+^	1.00×10^−2^	1.18	1.42	1.20
	1.00×10^−4^	1.13	1.38	1.22
	1.00×10^−6^	0.97	1.33	1.37

Ca^2+^+BS	1.00×10^−2^	1.47	1.42	0.97
	1.00×10^−4^	1.27	1.34	1.06
	1.00×10^−6^	1.11	1.23	1.11

(3) Detection of Ca^2+^ by CA(G) fixed on an electrode.				

**Measured substance**	**Concentration of substance [molL^−1^]**	**Electrode voltage [V]**		**Ion response**

		**State of an electrode surface**		

		**CA(G) free**	**Fixed CA(G)**	

Ca^2+^	1.00×10^−2^	1.18	1.20	1.02
	1.00×10^−4^	1.13	1.17	1.04
	1.00×10^−6^	0.97	1.09	1.12

Ca^2+^+BS	1.00×10^−2^	1.47	1.30	0.88
	1.00×10^−4^	1.27	1.18	0.93
	1.00×10^−6^	1.11	1.15	1.04

**Table 4.** Results of molecular orbital (MO) calculations.

**Acceptor chemical** ^(1)^	**Calculated MO value (eV)**		

	**HOMO**	**LUMO**	**Δ(LUMO-HOMO')** ^(2^,^3)^

CA(G)	−9.893	0.498	9.388
CA(B)	−6.303	1.412	10.302
MG	−8.660	-0.651	8.239
FE(N)	−8.947	-0.965	7.926
FE(B)	−8.351	-0.199	8.691
CU(I)	−8.141	-0.211	8.679
CU(II)	−5.376	-3.418	5.472

(1) Details are shown in Table 1;

(2) HOMO' value of the NH_2_-R-(SiO_2_)_n_ (R=-C_2_H_4_-NH-C_2_H_4_-, n=5) cluster was calculated as the model surface structure;
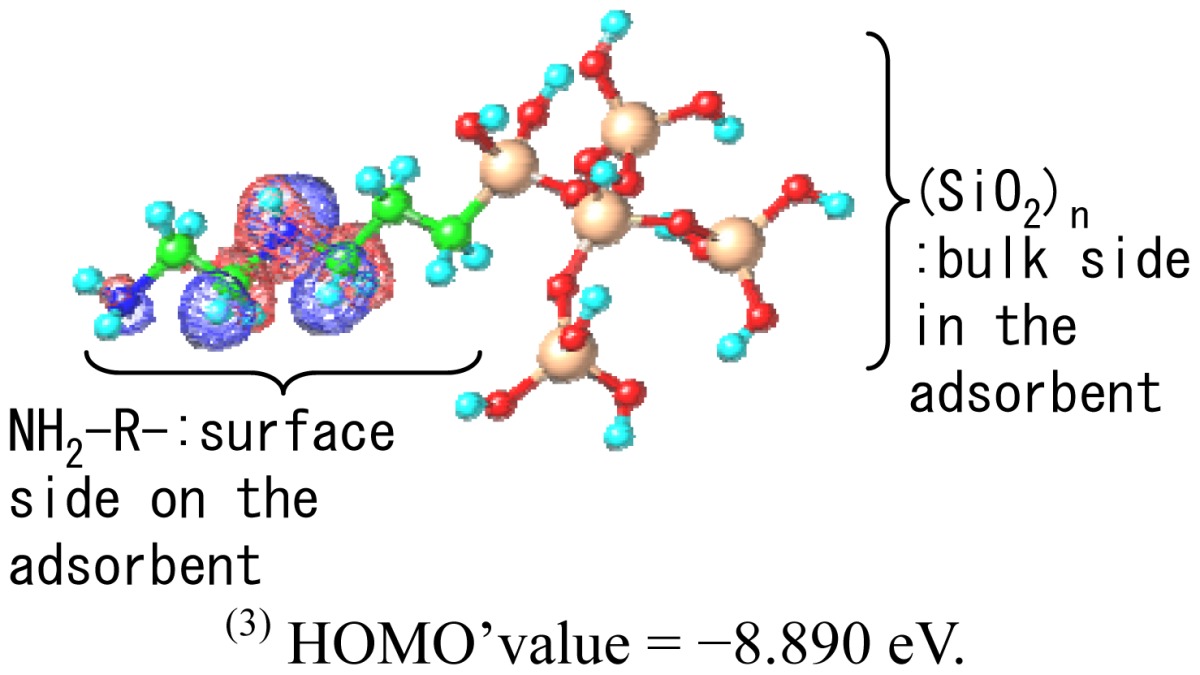

(3) HOMO'value= −8.890 eV.
